# Observations on the Surgical Pathology and Treatment of Aneurism

**Published:** 1841-10

**Authors:** 


### ART. X.

*Observations on the Surgical Pathology and Treatment of Aneurism.* By WILLIAM HENRY PORTER, A.M., Professor of Surgery in the Royal College of Surgeons in Ireland, Surgeon to Meath Hospital, &c. &c. —*Dublin*, 1841. Part I. 8vo, pp. 214.

THE high professional character of the author of this treatise, the extensive opportunities for practical investigations which he is known to possess, and the reputation he had already obtained in the surgical treatment of aneurism, made us hail the publication of his little work with much satisfaction. The subject of it is one of the most difficult and perplexing that engages the practical surgeon; we ourselves have experienced this in common with our brethren; and we cherished the hope that Mr. Porter, as one of the great authorities of the day, might be able to solve many knotty points, and set at rest many questions respecting which, from our own more limited opportunities, we felt unable to come to a satisfactory decision. That Mr. Porter has fully answered all our expectations and wants we can scarcely admit; yet is his work replete with valuable observations, and is characterized throughout by good sense, great discrimination, and sound conclusions. We have been much pleased with the clearness and precision with which the author has stated his own opinions and views, as well as those of others; and have especially admired the decided and reasonable course of practice which he has recommended, in various cases of the most interesting kind to the practical surgeon, whether among those requiring the prompt decision of the moment, or admitting of cool and deliberate investigation. The work everywhere gives evidence that the author has not followed blindly in the footsteps of others, nor jumped to conclusions on imperfect testimony; and that in framing his code of instructions he has not had reference solely to his library-shelves, but to the combined experience of his predecessors, and that derived from his own ample resources.

Though it must be allowed that in many professional subjects the British practitioner has been fairly eclipsed by his continental contemporary, it may be asserted, with all due respect to the labours of others, that the British surgeon has in a great measure rendered the subject of aneurism peculiarly his own. The scientific principles on which John Hunter's operation is founded, its originality, and its vast importance, have long since been conceded; and the fact cannot be denied that the British surgeon has led the way in all those bold and enterprising operations on the arteries for the cure of aneurisms which were previously, and but too truly, considered fatal and incurable diseases. The femoral arteries, the iliacs, the carotid, the subclavian, the innominata (for we can scarcely look upon Mott but as a British surgeon), and the aorta itself have all been tied for the first time by British surgeons; and though their example has been boldly and promptly followed by their contemporaries in different parts of Europe and America, we have just reason to feel proud of the laurels they have won for British surgery.

Since the year 1785, when Hunter first put his operation into practice, the press in this country has afforded abundant evidence of the great attention paid by our surgeons to the subject of aneurism and its treatment; in proof of which we need merely to repeat the names of Home, Abernethy, Cooper, Ramsden, Travers, Lawrence, Crampton, Hodgson, Norman, &c., and, though last not least, of Jones. The publications of Deschamps, Maunoir, Pelletan, Roux, Dupuytren, Velpeau, and many others, bear ample testimony to the industry and splendid talent bestowed on the same subject by our continental brethren. Of all the works, however, on aneurism, whether published on the continent or in this country, none have become such standard authorities as those of Scarpa and Hodgson. Since the appearance of the last, in 1815, no separate treatise, we believe, from any high authority has appeared, if we except the little volume of Mr. Wardrop (1828), and the more elaborate production of Mr. Guthrie. The former work had no pretensions to treat of aneurisms in general, but was in a great measure confined to the elucidation of the operation of Brasdor or Desault, and the modification which its author himself originated. The excellent treatise of Mr. Guthrie, however, comprised a brief history of all that had been hitherto known or done on the subject.

The profession have already been partly in possession of Mr. Porter's lectures, as they appeared in the Dublin Medical Press; we are happy, however, that he yielded to the request made to him for their separate publication, as they will be thus more extensively useful than they could have been in the pages of a journal. The part now published is only one half of the intended work; its object is thus stated by the author in his preface:

"In the present volume my object is to treat of aneurism in its generic forms (as far as is practicable), independent of the proper and peculiar features that may attach to it in any particular locality—to describe the different pathological conditions that precede and are supposed to conduce to the formation of the disease—to connect these with the symptoms as they arise—and to point out the mode of treatment apparently suitable to each variety. Another object in which I am deeply interested is, to endeavour to ascertain how far our curative measures may be insufficient and our operations unsuccessful, and to explain (to the extent our pathological information will permit), the causes and circumstances that thus exert an unhappy influence on our practice." (pp. viii-ix.)

In noticing the circumstances likely to conduce to the natural cessation of arterial hemorrhage, our author refers to an experiment of Jones's, which bears on a point of much practical importance hitherto little noticed by writers on the subject. In his sixth experiment Dr. Jones found, "that a teacupful of blood, taken immediately after the division of an artery, coagulated in five minutes and a few seconds. The same quantity, taken a quarter of an hour afterwards, (by which time the animal had lost an immense quantity of blood, and appeared very faint,) coagulated in three minutes and a half. Thus it appears that in proportion to the loss of blood, and the imminence of the danger, is the tendency of that fluid to coagulate increased, and the chief, if not the only measure that is eventually to preserve life furnished when most required." We much fear that any hope, grounded on this experiment, would prove fallacious, for, without at present taking into account the different circumstances under which the blood is placed when out of the body and when circulating in its natural channels, the results of some of the cases of aneurism which we ourselves have had opportunities of observing, lead us to conclude that great depletion is not productive of such advantages in this particular respect, as we might, *à priori*, be led to expect from it; nay, quite the contrary opinion is occasionally entertained, namely, that as the quantity of blood in the body is diminished, it becomes thinner, and hence less coagulable; and some even go so far as to imagine that, under Valsalva's treatment, the fibrin is so lessened in this fluid by the spare diet of the patient, whilst at the same time the heart's action becomes so irritable and increased in rapidity, that actually the opposite results from those expected are obtained. Certain it is that we, in common with most surgeons, have often seen the abstraction of blood in aneurismal cases, whether taken in large quantities occasionally or by small and oft-repeated bleedings, and whether combined with the most rigorously spare diet, or with a reasonable proportion of animal food, equally unsatisfactory in producing coagulation. That the reverse has been the result from time to time we admit, but then we also often see cases where large laminæ of fibrin have been deposited without any interference on the part of the practitioner. Mr. Wardrop, we believe, entertains the opinion, that the smaller the quantity of blood in the body the greater is its disposition to coagulate; but we cannot help thinking that one of his own cases, that of Mrs. Denmark (which however, be it observed, he does not bring forward to illustrate this subject), might be adduced as an example to the contrary.*

The whole of this question, particularly with reference to the treatment of aneurism, is well worthy of further investigation, and we could have wished that Mr. Porter had said more upon the subject.

* Cyclopædia of Practical Surgery, vol i. p. 231.

On the process by which hemorrhage is naturally suppressed and a wounded artery permanently healed, Mr. Porter succinctly states the best established doctrines in language not to be misunderstood; but we wish we could say the same of his statements on the question, whether a wound of an artery ever closes and leaves the canal of the vessel entire. At p. 141 Mr. Porter records his belief that "arteries are wounded in the operation of phlebotomy much more frequently than is imagined, and that many a patient has thus been injured without ever having been aware that he had been exposed to so imminent a peril." We wish much that we had been favoured with the author's grounds of belief, for, in our own experience, we have never met with an example of the kind; we have seen some instances where the evil effects have been of trifling extent, but the patients have always been aware that something unusual had occurred; and, indeed, were we to believe with the author (p. 19), that "a wounded artery never heals unless by obliteration," it appears to us altogether improbable that such a change could go on in a patient's arm, whether spontaneously or under the means employed by the surgeon, without his being conscious of it. In the first of these quotations (p. 141), we are left uninformed as to the condition of the vessel after injury, unless we conclude that the second (p. 19) reveals it; and if it does, the statement is irreconcileable with the impression conveyed at p. 18; for here he observes, that "a case of aneurism at the bend of the arm, and produced by the puncture of a lancet in bleeding, was treated by compression in Stevens's hospital, and recovered, *the artery remaining perfectly pervious through the entire of its course.*" Some doubts seem to have been entertained as to the nature of this case, but the quotations show that our author's views, or at least his statements, are not free from confusion. Our impression as to his opinion is, that he is doubtful if a wounded artery ever heals unless by obliteration; we ourselves are decidedly of opinion that it rarely does, but agree with him when he states that there has not been a sufficiency of evidence collected to decide the question in a satisfactory manner. The question is, however, one more of curiosity than of much practical import; for, in the arm, for example, when the injury is inflicted, the surgeon applies his pads and bandages with the sole object of suppressing hemorrhage, feeling perfectly indifferent whether, in attaining it, the canal of the vessel is obliterated or still left permeable.

The surgical treatment of hemorrhage has been, we think, too hurriedly passed over by our author. Pressure by means of compress and bandage, or by instruments; the ligature, torsion, styptics, the cautery, are all rapidly glanced at; we need scarcely say that the author gives his voice in favour of the ligature; but he is not by any means a bigot, and where circumstances are favorable, recommends a trial of other methods. We have often wondered that, in these days when novelty is so much the fashion, torsion has not been more extensively tried in this country. The practice, however, does not seem to be gaining ground among our hospital surgeons, notwithstanding the favorable reports of it from the continent; and for our own part we must confess our partiality to the ligature.

We wish that Mr. Porter had given something more on the subject of styptics; and we should have been pleased if he had raised his voice against the quackery which, from time to time even in these days, attempts to palm upon simple or unthinking members of the profession, some specific fluid, "warranted" (as the advertisements say) to restrain the flow of blood from such vessels as the carotid or femoral. We hold it to be the duty of every teacher to warn his pupils against reliance on any alleged specific in such cases; and to give them clearly to understand that no styptic employed to suppress hemorrhage from a large artery has ever yet, from the time of Warner downwards, produced any effect without the aid of pressure. It is worthy of remark that Mr. Porter attributes the efficacy of the cautery, styptics (for of course certain styptics are specific against oozings and small wounds), torsion, and ligature all to pressure; and we cannot question the reasonableness of his views, though we confess that we were in some measure struck with the novelty of the arrangement.

The observations on "pressure by means of compress and bandage, or by mechanical instruments," are so much to the point, and so characteristic of the author, that we cannot refrain from quoting them in full:

"Whatever predilection," says he, "a surgeon may entertain for the ligature, he nevertheless will meet many cases in which he will be disposed to try compression, and some in which he can have no other resource. Thus, his business may be with a patient who has an unconquerable dread of the knife, and who will steadily refuse to submit to those incisions that are almost always requisite to expose a wounded artery, so that it may be tied with safety. Or the wounded vessel may lie deep, or it may be of such size as to encourage a reasonable expectation of the bleeding not proving very profuse; or there may be nerves, or other very important parts liable to be injured; or the accident may have happened at night, and in a situation where it might be impossible to procure light and the assistance necessary to the performance of a very delicate operation. I pass over cases where there may be reason to suspect a diseased condition of the vessel, and the still more dreadful cases of secondary hemorrhage, where the ligature had been already tried and failed. Independent of these, the recent cases are numerous, in which a surgeon is obliged to trust to compression and bandage, and the success that occasionally attends this practice will appear surprising when the difficulties and dangers that accompany it are considered.

"If we reflect on the subject physiologically, it appears necessary, in order to the perfect compression of an artery, that the vessel should be small in size and situated superficially, for if a strong muscle intervenes, its contraction will be sufficient to raise the compress and allow the vessel to bleed internally. The artery should lie upon a bone, so as to afford a counter-resistance to the pressure from without; there should be no accompanying vein, nor indeed any vein in the neighbourhood through which the current of blood could be intercepted by the compress; there should be no accompanying nerve; in a few words, the artery should be small and healthy, superficial, insulated, and resting on a bone, and a very slight anatomical reflection will show how few the arteries are that are so fortunately circumstanced. Even in the most favorable cases, there are still some apparent difficulties. The compress and bandage ought to be applied so tightly as to lay the opposite sides of the wounded vessel fairly in apposition, and in general this occasions such a degree of pain that few patients (once the actual terror of bleeding to death has passed away) will, or even can, endure it. All bandages will stretch and become loose, the patient will shift about and endeavour to alter its position and its bearing, and I have known one who actually took it off. If the bandage is too loose, it may not control the bleeding, or, what is worse, preventing it from appearing without, it may permit the vessel to bleed internally, and in a few hours the surgeon, summoned by the agonizing distress of his patient, finds the limb swollen, its cellular tissue in every direction injected with blood, and the entire ready to fall into gangrene. If, on the contrary, the bandage is too tight, and particularly in persons of bad habit and debilitated constitution, the pressure necessary to control the bleeding may occasion mortification of all the structures beneath it. Of all the arteries in the body, the temporal has been justly considered to be that which may with most safety be entrusted to pressure, yet even here very calamitous results are by no means improbable. Soon after the application of the bandage, the patient complains of intense pain; and when the dressings are removed, the integuments are found in a state of erisypelatous inflammation, threatening to run into gangrene, the wound opening, and the blood bursting out again; when (as I have unfortunately witnessed,) the compression is reapplied, it only hastens the mortification without at all controlling the bleeding, and nothing that the patient can endure will restrain the hemorrhage, which is profuse, and often periodical, until that condition of constitution is established, known by the appellation of hemorrhagic fever; at last, perhaps, the trunk of the artery is tied at a distance from the slough, and such an operation may succeed, but, in consequence of the freedom of anastomosis among the branches, is far more likely to fail. Thus harassed and broken down, pale and exsanguine from repeated losses of blood, either the patient dies at an early period, or the foundation is laid for dropsy, consumption, or some other disease that will eventually carry him off. This is a sad picture of events, and unfortunately it is sometimes a true one, but not by any means so frequently as a mere theoretic reasoning on the subject might lead us to believe: in practice we find the treatment by compression tolerably successful, and that too in situations and under circumstances that might not always be anticipated.

"When compression is resorted to for the purpose of controlling hemorrhage, a graduated compress is to be placed exactly over the aperture in the bleeding vessel, and secured there by a bandage rolled so evenly, that no one part of the limb is subjected to greater pressure than another, and so firmly as to lay the opposite sides of the vessel fairly in contact, but at the same time occasion as little pain as possible. This is generally accomplished by a comparatively moderate degree of pressure, and I believe the important point to be attended to is the evenness of the bandage rather than the force it exerts. In the arm, for instance, which is frequently the seat of this operation, in consequence of the artery being punctured by an awkward and ignorant phlebotomist, the bandage should commence with the fingers, each of which should be rolled separately and firmly; it must then be carried evenly up the arm, and terminate a short distance above the position of the compress. The arm should then be laid upon a pillow, and the patient receive the strictest injunctions not to attempt to move it. For some hours the case will require the most watchful attention on the part of the surgeon. If there is no increase of pain, or throbbing, or other uneasiness, the compression will probably succeed, and I believe many an individual has had the brachial artery opened without ever entertaining a suspicion that he had been exposed to so much peril: if there is this increase of pain or tension, the arm must be opened and examined lest bleeding should be going on internally." (pp. 21-5.)

Aneurisms have been classified by our author, according to the usual doctrines of the day, under the heads of *true*, *false*, and *mixed; circumscribed*, *diffused*, and *traumatic;* the *aneurismal varix*, the *varicose aneurism*, and a particular form of the disease which has been described by him at greater length in the first volume of the Cyclopædia of Anatomy and Physiology. We are pleased with the general distinction Mr. Porter draws between dilatation of the whole circumference of an artery, and that at one or other side of the vessel, which latter really constitutes what a surgeon of the present day understands to be an aneurism. We are induced to refer to this point, as we occasionally find even now, when pathology is so much cultivated, that with some of our friends the value of the distinction does not seem to be sufficiently appreciated. A dilatation or enlargement of the caliber of an artery in its whole periphery, even though it should bulge a little on one side, does not constitute aneurism. We have watched such dilatations of the aorta, innominata, and other large vessels for years, and have observed no marked changes in them. On one occasion we were actually in consultation on a case of this kind, in which it was in contemplation to tie the iliac artery. There certainly was a wonderful throbbing in the common femoral, but the vessel on the opposite side was in a similar state, and so were most of the other large arteries. The surgeon's attention had been attracted to one side only, in consequence of an injury to the hip: we watched the case for a very long time, but could perceive no change, nor could we observe that the patient was in the least inconvenienced by this condition of her arteries.

We have often contemplated an attack upon our present nomenclature as regards aneurisms, and wish we could have taken the field in company with Mr. Porter: he seems, however, to prefer the terms as used in the schools of the day; and perhaps, for the sake of his pupils, he has been unwilling to make the subject appear more complicated than it actually is. To some of the names we make no objection; but we hope that Mr. Porter agrees with us, that the terms *true*, *false*, and *mixed*, are most objectionable. In many instances, even at a *post-mortem* examination, they cannot be applied with propriety, and certainly much less so on the living body; even if correctly applied, the distinction is not of the smallest practical utility; the disease in the one case being attended with no particular symptoms; being equally formidable as in the other; and, in all, requiring exactly the same kind of treatment, the result of which is in no shape, unless under very particular circumstances, influenced by the condition of the vessel.

"There is a pathological condition of an artery producing all the symptoms of circumscribed false aneurism, and apparently cureable by the same means, which, as yet, has only been noticed and described by myself. It seems to be formed by a dilatation of the fibrous and cellular coats, and the absorption of the internal lining. It appears to be so far a true aneurism as that the artery is uniformly dilated around its entire circumference, and it is so far a false one, that the internal lining membrane has been removed." (p. 34.)

We are convinced that such examples are occasionally to be met with, and we have some in our own possession which we have been accustomed to refer to, not so much for the purpose of illustrating Mr. Porter's form of the disease, as to show the folly of limiting the component parts of an aneurismal sac to one, two, or three of the tunics. In one instance it is impossible to say whether the serous tunic of an artery lines a sac or not; in another, although we are satisfied that it cannot have stretched so far, we however feel unable to say where it has given way, or whether the middle coat ceases at the same point, extends somewhat further, or even lines the greater part of the sac; then again in some examples there can be no doubt that the sac is mainly if not entirely formed by the outer tunic, whilst in other cases it is impossible to believe that this tunic has still continued to form this part. It suffices to instance aneurisms of the lower part of the thoracic aorta which have made their way to the surface of the body, and which are so common in our museums. But lest it should be objected that these are internal aneurisms and in many respects different from the cases in which the surgeon takes a therapeutical interest, we shall refer to a preparation in our possession of an aneurismal tumour of the superficial femoral artery, about the size of a large orange, which during life presented, as Mr. Porter would say, all the symptoms of circumscribed aneurism, but whether it was *true*, *false*, or *mixed* we must confess we made no attempt to ascertain, being unacquainted with any diagnostic difference for aneurismal tumours of such a size. After an unsuccessful operation the tumour came under our inspection, and a large part of the circumference of the sac was found to consist of consolidated muscular fibres; portions of the sartorius, vastus internus, &c., so mingled up with cellular texture and newly-deposited lymph, as to give it (the sac) the appearance of an old and thick cyst of an abscess; and what made it impossible that in this case the dilatation of any tunic or of all these could have existed was, that the aperture between the artery and the sac was something less than half an inch in diameter. The parts were much in the condition of some forms of *traumatic* aneurism, yet it had been of spontaneous occurrence.

"When there are two or more idiopathic aneurisms present," (says our author, p. 36,) "it exhibits such evidence of unhealthy condition of the whole system, as to hold out no reasonable expectation of recovery, and confines us to the employment of palliative measures alone." Such a condition should in our opinion demand a careful investigation on the part of the surgeon, but ought not to be looked upon as so utterly irremediable. It is to be hoped that Mr. Porter does not, unless there be positive proof of an internal aneurism, hold out such melancholy prospects to his patients; and we suspect that he must occasionally gainsay his own *dictum*, for we find at p. 118, "in March 1835, a man was admitted into the Meath Hospital with popliteal aneurism in each ham," on whom an operation was sanctioned; and, though the patient died sixteen days afterwards, there is no proof that death was the result of the double aneurism; but on this point it would have been desirable that Mr. Porter, for the sake of his pupils, had not been so dogmatic; for there are numerous cases on record where patients have been cured of double aneurisms by operations. Mr. Guthrie, for example, (p. 61,) refers to a man whom he knew, who survived the operation for popliteal aneurism on both limbs for twenty-five years; and Sir Wm. Newbigging (Edinb. Med. and Surg. Journal for Jan. 1816) cured an inguinal and popliteal aneurism at the same time by tying the external iliac; but we need not multiply such examples, as we trust they are sufficiently known already.

Our author's observations on the alleged causes of aneurism (p. 37 et seq.) are worthy of note for their good sense; he seems to hint at inflammation being occasionally a cause, but if it is, the occurrence, in our opinion, must be rare.

On the symptoms of the disease (p. 53 et seq.) we have nothing new, unless it be that the crooked and stiff condition of a limb may sometimes lead to suspicion. Such a state has been observed by Mr. Porter in the fingers, wrist, and elbow, in axillary aneurism; the toes and foot he has also known affected in popliteal aneurism, but we doubt if such conditions are truly characteristic of the disease. We have at present under our charge a young female with abscess in the ham, with the toes, foot, and knee crooked and stiff, and have seen many of a similar kind. In bubo of the groin or armpit similar effects are seen, and though we do remember a patient of ours with a popliteal aneurism complaining for the first time to us of pain and stiffness in the ankle, we must say that we place no value on the observation. Mr. Porter is not inclined to consider the *bruit de soufflet* as pathognomonic, and we heartily concur with him; we have lately had under our charge two cases of supposed aneurism, in which the sounds were exactly similar, and on dissection, one proved to be dilatation merely, whilst the other was an aneurism the size of a turkey's egg.

At pp. 63 and 132 some judicious observations will be found on the difficulty of diagnosis in thoracic and in diffused external aneurisms. The stethoscope is very properly recommended, but is shown to be only of limited value in such cases.

Our limits will not permit us to follow Mr. Porter through the remaining portions of his observations on internal aneurism, but we recommend to notice a case supposed to be laryngitis, in which our author cut into the trachea, though with no permanent benefit, as the disease was aneurism of the arch of the aorta. We ourselves have seen something of the same kind in aneurism of the innominata, where the real disease was not even suspected. We cannot but admire the candid manner in which Mr. Porter admits his mistake in this case. Such ingenuousness elevates him in our estimation; were all surgeons equally candid the younger branches of the profession would have less to learn for themselves.

In the section on Treatment there is no affectation of novelty; no views or plans which our author claims as peculiarly his own; yet there is, as usual, much good sense and judicious discrimination; and there are very few points in which we are disposed to differ from the author. Inflammation of the sac he considers a very rare spontaneous cure, and we are of the same opinion; gangrene with sloughing he deems equally rare; pressure by the tumour on any part of the artery above or below he admits may produce obliteration, but, he truly adds (p. 76), "in the majority of cases, we seldom see one of them exemplified." To this list of spontaneous causes he might have added displacement of a mass of fibrin or of the entire lamina, for we are convinced that this must occasionally be a spontaneous cause of cure, that is, a cure without the particular interference of the surgeon; and we could refer to cases illustrative of our opinions which have occurred under our own observation.

Amidst a profusion of valuable practical observations in this part of the work, the following seems to us so interesting that we cannot forego the opportunity of adding to its publicity. In December, 1831, Mr. Porter attempted to pass a ligature round the innominata for the cure of a very large subclavian aneurism, but failed in consequence of the vessel being extensively diseased. The aneurismal tumour entirely disappeared; the patient's health and strength were so completely restored, that he was able to resume his occupation as a day-labourer, and he is alive and well at the present date. Mr. Porter knows not whether to designate this cure as spontaneous or otherwise.

The following passage, we think, ought not to be passed over without comment:

"At first, I imagine the process by which aneurism is cured by the applica-

**tion of a ligature at its cardiac side, is not the same in the false aneurism and in the true; at least it is difficult to explain the varieties met with on any other supposition. In the false the great object to be accomplished is, the removal of the impulse of the heart from the blood contained within the sac for a sufficient time to allow of this reservoir becoming slowly and gradually filled with blood, and for that blood to become firm and coagulated. This coagulum, then, is pressed upon or against the wounded and bleeding vessel, and produces those consequences within it that terminate in its obliteration; the blood, if the case proceeds favorably, is afterwards absorbed, and the sac, in process of time, is converted into a solid piece of ligamentous substance similar to that into which the arterial trunk has degenerated. The completion of this process requires different periods of time, according to the size of the vessel, the dimensions of the sac, and the quantity of fluid blood it can contain, but is always accomplished long before the separation of the ligature, and therefore, if there could be any other means devised for removing the impulse of the heart during the required period, a ligature need never be applied for the cure of aneurism." (p. 110.)**

We feel assured that Mr. Porter cannot have bestowed much attention to the composition of this passage, else he would never have deviated so far from his usually accurate style, nor allowed to appear under his sanction such a tissue of misapplication of terms, inaccuracies as to facts, and illogical deductions as are comprised in the above quotation. In the first part of it we are told that the process of cure after the application of a ligature is not the same in *false* aneurism and in *true;* now, as these terms are generally understood we know of no shade even of difference; the process as described in reference to the *false* appearing to us as being equally applicable to the *true.* We are much mistaken, however, if in this passage Mr. Porter does not refer to that kind of *false* aneurism termed *traumatic,* in which case "all the symptoms of circumscribed false aneurism," we admit, might be made out, but even allowing the case to be traumatic, if a ligature is applied on the cardiac side of the disease, and the cure proceeds favorably, we cannot see any distinction of importance in the subsequent phenomena. We know not our author's meaning when he talks of the operation giving time for "this reservoir (sac) becoming slowly and gradually filled with blood," for an empty aneurismal sac is what we suppose never exists in the living body: if by "blood" clot or fibrin is meant, then could we make allowances; but the after-statement "and for that blood to become firm and coagulated" removes all doubt of the author's meaning on this point. Again, the assertion that the conversion of the sac and artery into a "solid piece of ligamentous substance is always accomplished long before the separation of the ligature" is, as Mr. Porter must well know, not consistent with fact. Let us refer to what may be considered the progenitor of all such preparations, viz., that in the collection at the College of Surgeons, London, which illustrates Hunter's first case, taken from the person fifteen months after operation; even at this distant period the sac is still distinct, and we have seen similar conditions many years after successful operations. The ligature from a main artery may separate at ten, twenty, or thirty days, and we will not task the patience of our readers by an attempt to prove , what must be plain to all, that the subsequent alterations in an aneurismal sac, and the main artery connected with it, must be the work of a longer period than the few days Mr. Porter's remarks would lead us to suppose. Perhaps it is meant that the ligature, ere it separates, produces those consequences which lead to obliteration and conversion into a solid ligamentous substance, but it will take some ingenuity to give the passage above quoted this meaning. The process for the completion of the cure, says Mr. Porter, "is always accomplished before the separation of the ligature, and, therefore, if there could be any other means devised for removing the impulse of the heart during the required period, a ligature need never be applied for the cure of the aneurism," a conclusion exactly the reverse of that we should draw; for, had we stated as much for the effect of the ligature, we should have said "and *therefore* if other means could be devised for removing the impulse of the heart during the required period, unless these means were proved to be decidedly superior, the ligature should be preferred to all." We object not to the intended hint that if other and better means could be devised, &c., the surgeon should prefer them, but we doubt if Mr. Porter's deduction from his premises is consistent with the rules of logic.

We make these remarks with most perfect good will towards Mr. Porter; and it is quite foreign from our intention to cavil maliciously at either word or expression; we have stated what we imagine to be the real meaning of the above passage, and should be sorry to find that we have misunderstood it.

It appears, from the work before us, that Irish surgeons have had no experience in Brasdor's or Wardrop's operation; and in consequence, Mr. Porter, with his usual good taste, says little on the subject; he however declares his conviction that such operations should be resorted to in cases deemed favorable, and when Hunter's method cannot be followed out, which is all that its warmest advocates claim for the proceeding. It is highly desirable, in the state in which this question has been left, since the publication of Mr. Wardrop's article on aneurism in the Cyclopædia of Practical Surgery, that some one enjoying opportunities such as those possessed by Mr. Porter, should take a prominent part in giving a just estimate of these proceedings. Mr. Guthrie, in the work to which we have already referred, is the only English author who has bestowed much consideration on the subject; and without adverting to the manner in which he has done it, we shall only say that our opinions do not entirely coincide with his, nor has time proved that he was right in some of his most important conjectures.

We have perused the section on *diffused* aneurism with less satisfaction than some of the others. In the first place we cannot agree with Mr. Porter in some of his distinctions between *diffused* and *traumatic* aneurism; nor can we admit the practical utility of the distinctions drawn between the two kinds of traumatic aneurism which he refers to. If, as is supposed by Mr. Porter in illustration, an artery is ruptured by external violence, though there be no external wound, or is punctured by a sequestrum in necrosis, we see no good reason for refusing to adopt the word *traumatic*. If a person has a compound fracture of the foot or leg, causing gangrene even as high as the thigh, we call it *traumatic* gangrene; all that the term implies being that the gangrene is the result of a wound. We are still less inclined to agree with the following statements:

"An external wound so leading down to, and communicating with, the injured artery as to permit the escape of a portion of the blood from it,........ is the essential condition of traumatic aneurism. Thus, the affection may have been produced by a wound, yet if that wound is healed, or not being healed, if it is very oblique, or if there is any circumstance that prevents the blood from passing out of the limb or part, it falls not under the appellation of traumatic aneurism. It is necessary to be precise on this point, for it *seems* to be a misapplication of the term, the word 'traumatic' being generally used in surgery to imply that the symptom or condition referred to has been the result of external violence; here, however, it is much more limited, and is confined to one particular class or condition of injury. Thus, a student, in bleeding a patient in the arm, may puncture the artery and an aneurism be the consequence, yet if the wound heals, it is not a traumatic aneurism nevertheless, the latter condition implying the existence of a wound through which fluid blood might trickle or a coagulum protrude." (pp. 128-9.)

That such distinctions are not drawn by the professsion we are firmly convinced, and they are too arbitrary to influence us. Pads and bandages may, and indeed often, " prevent the blood from passing out of the limb or part," yet the effusion of blood from the wounded artery is not the less traumatic aneurism, and the mere circumstance of the puncture of the skin in phlebotomy having healed or not makes, in our opinion, no great distinction; the cutaneous wound cannot heal before the lapse of some hours, yet it would be absurd to apply the term traumatic aneurism before union, and immediatety after to say that the case is not traumatic aneurism; and as for the additional distinction that the true traumatic aneurism implies " the existence of a wound through which fluid blood might trickle or a coagulum protrude," we maintain that it is equally useless. In drawing these distinctions, however, Mr. Porter seems to have this practical conclusion in view, that, in what he terms the traumatic aneurism, as the blood may still be conveyed through the wound in the skin by the collateral circulation, even after Hunter's operation has been performed, it is the most correct practice to cut into the swelling, turn out the clots of blood, and secure the bleeding orifices. We cordially agree with him here, but dissent from the practice inculcated in other cases, in which, after judicously recommending a trial of pressure, he seems to advocate the application of the Hunterian operation. Even though the wound may be closed on the surface, the collateral circulation, it appears to us, may still cause an increase of the internal bleeding; and as we are not so deeply impressed with the difficulty and danger (either at the moment or subsequently) of cutting into such collections, as Mr. Porter seems to be, we hesitate not to recommend this proceeding as the practice most likely to be attended with favorable results; and indeed we must express our surprise at our author's apparent reluctance to cut into such collections, when we find him, regardless of the danger from exposure and inflammation, in another part of his work advocating the practice of cutting freely into aneurismal tumours when suppuration has ensued after operation. It is to be observed, however, that these remarks of ours are chiefly applicable in the case of wound during venæsection, the instance most frequently referred to by our author himself.

Among the various causes of failure of operations for aneurism, and " to show what is already but too well known to every one conversant with the history of surgery, that operations, even when well planned and well executed, sometimes fail," Mr. Porter has brought forward a number of interesting cases and facts in illustration of his views. He enumerates many of the usual and acknowledged obstacles to a successful result; but we need not go over ground already sufficiently well known. We cannot help, however, referring the reader to some singular observations on what Mr. Porter terms "reflex action of arteries."

Perhaps the best section in the volume is that on Secondary Hemorrhage, and we recommend it most particularly to our readers. Although we do not even here accord with the author in every point, we have been struck with the soundness of the principles everywhere conspicuous; and we hardly know which to admire most, his valuable doctrines or the modesty with which he propounds them.

The author first takes some notice of the causes considered likely to induce the frightful result of secondary bleeding, and then states what he considers best to be done under the circumstances. He clearly proves that neither considerable disturbance and denudation, nor the application of a ligature near to a collateral branch are of necessity followed by this result, but, notwithstanding, he duly cautions the surgeon against negligence on these points; he seems to imagine that he can foretell the coming on of hemorrhage by certain symptoms (p. 179), and he asserts that on all occasions the blood flows from the distal end of the artery. We give him credit for referring to this last point, as we are convinced that until within these few years the reverse was generally supposed, yet we cannot go the whole length with him; for if this were "always" the case, it would be difficult to account for secondary bleeding after amputation. He ingeniously endeavours to prove the existence of two kinds of inflammation in an artery near the seat of deligation, *healthy* and *unhealthy*, the former being immediately above the ligature, the latter below; and hence he ascribes the frequency of secondary hemorrhage at this end of the vessel. But unfortunately the only case he brings forward in proof is one in which secondary bleeding did not occur. We are not therefore convinced of the truth of this singular view given by Mr. Porter; and from a most careful examination of the artery, in several cases which have happened in our own practice, we must say that if such remarkable differences existed they were overlooked by us. From the consideration of these cases, however, and others that we have seen, we entirely concur in Mr. Porter's general assertion that unhealthy inflammation is the most frequent, perhaps the only cause of secondary hemorrhage; taking this latter term in the sense usually understood by surgeons, and meaning by the term unhealthy inflammation, merely what Mr. Porter means, viz. that nature shows little or no disposition to deposit and organize the quantity of coagulable lymph, which seems requisite to render the obstruction of the artery complete and permanent.

He next proceeds to examine the resources possessed by art, if any, "when the blood has burst forth." He objects decidedly to the plan of placing a ligature higher up on the artery, that is, as we understand him, at some distance from the original seat of operation.

"I will not dwell for a moment on the unreasonable nature of such practice, as long as there is a collateral circulation to convey blood to the open vessel; but merely enquire if the superior segment is closed and blocked up with lymph, or if, as I have before noticed, the ligature still remains upon it, whilst the lower had separated and was bleeding, of what possible utility could the tying of the artery higher up prove? It could only remove the impulse of the heart from the superior section, from which no danger is to be apprehended, but could have no influence on the lower, already deprived of that impulse, and supplied only by the collateral branches. If the operation, then, be useless, that would alone be a sufficient argument against subjecting an unfortunate patient to additional suffering and misery; but it is decidedly and positively injurious, and constantly aggravates his condition. I say 'constantly,' because, although a few solitary patients may have recovered after, or, more correctly, in despite of the operation, yet the numbers are so small as scarcely to constitute an exception to a general rule." (p. 191.)

We cannot here help thinking that Mr. Porter's judgment is warped by the prevailing idea that hemorrhage occurs *only* at the lower orifice; we have already given our opinion on this subject, but cannot avoid again reverting to the case of secondary bleeding after amputation. In the leg, for example, ligature of the femoral seldom (if it ever) fails to arrest the bleeding from the stump below the knee; and this is no imaginary case of collateral circulation, for we know that such vessels positively exist.

After some further remarks on the operation, on the necessity for pressure as an additional security, and on the attendant danger of gangrene, Mr. Porter proceeds to describe the treatment he recommends:

"On the appearance of the blood, the wound should be opened up, the granulations broken, and the mouth of the bleeding vessel fairly exposed; on it a small piece of prepared sponge should be placed deep in the bottom of the wound, care being taken that the pressure should be confined to this one spot; on this a small compress of lint, and another and another successively, until the graduated compress shall have attained a level higher than the adjacent surface of the limb. This must be firmly retained without being permitted to slip, and without allowing the escape of a single drop of blood; and, if this can be accomplished for three or four days, there is every reasonable probability of success. But here is the great difficulty. It is scarcely possible to apply a bandage, so as to answer the purpose, without interfering with the venous circulation and occasioning great pain; and as all bandages stretch and slip, and become relaxed, no certain reliance can be placed on them in a case where the patient's feelings encourage him to disturb them." (p. 194.)

Were the case ours, and did we resort to such bold practice as cutting down to the bleeding vessel, we should feel most inclined to place a ligature on each end of the vessel: we should, for the time, place more reliance on the thread than on pressure; there would be less immediate danger of gangrene, and we suspect as much ultimate chance of a permanent closure, as by pressure in the way recommended above. To be sure, it may be said, that as the ligature has failed at this part once already, why try it again? The case, we admit, is a most difficult one, but from what we have been able to learn on this subject, and what we have seen in our own practice, should we ever again be so unfortunate as to have similar cases to manage, we have made up our minds to try this proceeding. Pressure, amputation, or ligature higher up may afterwards be thought of, in the event of this method not succeeding. Of course, in some vessels, such as those at the root of the neck, no other plan than pressure can be followed.

But our limits will not permit us to follow Mr. Porter further at present; and we shall therefore conclude by thanking him for the information he has afforded us, and earnestly recommending his work to our readers. Mr. Porter has everywhere evinced a thorough mastership of his subject, and has stated his observations, his summing-up of the opinions of others or of his own, in a concise, unaffected, and generally clear manner. His style is not altogether free from faults; but for this the value of the materials makes ample atonement.

Mr. Porter has promised us a Second Part, and we confidently hope that he will not, like his distinguished contemporary, Mr. Colles, in the case of his Surgical Anatomy, give us reason for disappointment and regret that a work so well begun has not been completed. It is to be hoped, also, that ere long Mr. Hodgson will favour the profession with a new edition of his treatise; and should both works appear about the same time, we have laid out for ourselves a most agreeable and instructive task, in again bringing under the notice of our readers the pathology and treatment of aneurism.

---

